# Categorisation of EEG suppression using enhanced feature extraction for SUDEP risk assessment

**DOI:** 10.1186/s12911-020-01309-5

**Published:** 2020-12-24

**Authors:** Juan C. Mier, Yejin Kim, Xiaoqian Jiang, Guo-Qiang Zhang, Samden Lhatoo

**Affiliations:** 1 Department of Chemical Engineering, University of Houston, Houston, TX USA; 2 Department of Computer Science, University of Houston, Houston, TX USA; 3 School of Biomedical Informatics, University of Texas Health Science Center at Houston, Houston, TX USA; 4 Department of Neurology, McGovern Medical School, University of Texas Health Science Center at Houston, Houston, TX USA

**Keywords:** EEG, Machine learning, SUDS, Epilepsy, Feature engineering

## Abstract

**Background:**

Sudden Unexpected Death in Epilepsy (SUDEP) has increased in awareness considerably over the last two decades and is acknowledged as a serious problem in epilepsy. However, the scientific community remains unclear on the reason or possible bio markers that can discern potentially fatal seizures from other non-fatal seizures. The duration of postictal generalized EEG suppression (PGES) is a promising candidate to aid in identifying SUDEP risk. The length of time a patient experiences PGES after a seizure may be used to infer the risk a patient may have of SUDEP later in life. However, the problem becomes identifying the duration, or marking the end, of PGES (Tomson et al. in Lancet Neurol 7(11):1021–1031, 2008; Nashef in Epilepsia 38:6–8, 1997).

**Methods:**

This work addresses the problem of marking the end to PGES in EEG data, extracted from patients during a clinically supervised seizure. This work proposes a sensitivity analysis on EEG window size/delay, feature extraction and classifiers along with associated hyperparameters. The resulting sensitivity analysis includes the Gradient Boosted Decision Trees and Random Forest classifiers trained on 10 extracted features rooted in fundamental EEG behavior using an EEG specific feature extraction process (pyEEG) and 5 different window sizes or delays (Bao et al. in Comput Intell Neurosci 2011:1687–5265, 2011).

**Results:**

The machine learning architecture described above scored a maximum AUC score of 76.02% with the Random Forest classifier trained on all extracted features. The highest performing features included SVD Entropy, Petrosan Fractal Dimension and Power Spectral Intensity.

**Conclusion:**

The methods described are effective in automatically marking the end to PGES. Future work should include integration of these methods into the clinical setting and using the results to be able to predict a patient’s SUDEP risk.

## Background

3000 people die annually in the United States from Sudden Unexpected Death in Epilepsy (SUDEP), which has increased in awareness considerably over the last two decades and is acknowledged as a serious problem in epilepsy. SUDEP is defined as the sudden and unexpected, non-traumatic and non-drowning death of a person with epilepsy, without a toxicological or anatomical cause of death detected during the post-mortem examination. The definition lends itself to the fact that this phenomenon is not yet fully understood by modern medicine. SUDEP is death of an epileptic patient without any other explanation [1, 2].

The scientific community remains unclear on the reason or possible indicators that can discern a seizure that is indicative of a high risk for SUDEP later in life from other similar non-fatal seizures. Several risk factors are being investigated as candidates for risk assessment including the severity of seizures, non-adherence to treatment regimens, gender, genetic mutations and others. The duration of postictal generalized EEG suppression (PGES) is also a promising candidate to aid in identifying SUDEP risk.

PGES is a current area of interest and research in epilepsy. Patients who experience SUDEP are likely to have experienced PGES, Although not fully understood, PGES may be associated with a suppression of activity in the brain stem respiratory centers. this suppression of activity may lead to an inability for the brain to send signals to the lungs to expand and contract, leading to apnea.

Traditional EEG data analysis for the detection of the end of PGES is an intensive and manual process. Historically, labeling and detection requires trained physicians to inspect the data visually. This process is labor intensive, inefficient and subject to a increased variability as many times physicians disagree on the labeling of a segment of interest. The proposed method is a way of automating the detection of the end to PGES with decreased variability.

## Methods

To address the problem of automatic marking of the end to PGES, a machine learning architecture is proposed for EEG. In this architecture a broad feature extraction methodology is used to preprocess the raw EEG data. The extracted features are used to train one of two models, a Gradient Boosted Decision Trees algorithm (XGBOOST) and a Random Forest Classifier [3, 4].

### Data preparation

First the raw EEG data training set was processed. In a clinical setting, practitioners and subject matter experts participating in this research project agree that the end of PGES should be detected within 10 s. Therefore, the maximum window size that we allow is 10 s. However, the temporality of the data will be taken into account by creating 4 distinct training and testing datasets using the same data but with varying EEG data window sizes. EEG snippets of a constant 3 s, 7 s, 10 s, and datasets of random window sizes, one for random snippets of 1–12 s and the other of snippets of 20–30 s, were tested and compared. Each EEG data sample was labeled with a 1 or a 0 representing the presence of a state change in PGES within that window or snippet. In other words, the snippet samples were labeled with a 1 if they contained the end to PGES and a 0 otherwise.

The result of this sampling method was four rounds with each round containing a data set of  12,600,000, EEG snippets of 10, 7, 3 or random second EEG window sizes from 134 patients and represented by 10 channels which were then used to compute 10 distinct features described next.

### Feature extraction

Computer aided systems tackling classification on EEG data or other temporal data rely on characterizing a signal into certain features. EEG features obtained as a result of this feature extraction come from many fields of study such as: signal processing in the case of power spectral density, computational geometry in the case of fractal dimensions, information theory in the case of the different entropy implementations, etc. The EEG signals in the SUDEP data set are processed using pyEEG, an open source feature extraction tool originally designed for EEG time series data applied to diagnosing epilepsy in patients. Table 1 shows the features extracted from the EEG signals. This approach is rooted in the fundamental behaviors that trained professionals look for when manually analyzing EEG signals [5–8].

**Table 1:** Features extracted

Feature name	Return type	Category
Power Spectral Intensity	Two 1D vectors	Frequency domain
Petrosian Fractal Dimension	Scalar	Time domain
Higuchi Fractal Dimension	Scalar	Time domain
Hjorth Mobility and Complexity	Two scalars	Frequency domain
Spectral Entropy	Scalar	Time domain
SVD Entropy	Scalar	Time domain
Fisher Information	Scalar	Time domain
Detrended Fluctuation Analysis	Scalar	Time domain
Hurst Exponent	Scalar	Frequency domain

#### Power Spectral Intensity and relative intensity ratio (PSI)

The PSI is a measure of the strength of the signal as a function of frequency. It provides information on the strength of frequency variations. It is the magnitude of the squared Fourier Transform in a time series with a finite power signal.

The PSI is given by,

$$\begin{aligned} PSI_{k} = \sum _{i=\left\lfloor N(f_{k}/f_s) \right\rfloor }^{\left\lfloor N(f_{k+1}/f_s) \right\rfloor }\left| X_i \right| , k = 1,2,\ldots K-1 \end{aligned}$$

where, $\hbox {f}_s$ is the sampling rate, and N is the series length.

#### Fractal dimension

Fractal dimension comes from a branch of mathematics and it represents a ratio corresponding to complexity in a pattern. This ratio shows how a fractal scales differently from the space it is embedded and relates to the shape or fluctuations in time that is in a way self-similar. In other words the Petrosan Fractal Dimension a measure for the similarity of the whole EEG snippet to a proper subset of that EEG snippet. The fractal dimension can be found bu segmenting the signal into smaller sections and computing the number of self similar properties that comprises the original signal by amplifying the smaller section to fit the original signal .

*Petrosan Fractal Dimension* The Petrosan Fractal Dimension is one such implementation for calculating the FD in EEG time series data [5, 9, 10]. Its implementation is given by,

$$\begin{aligned} PFD = \frac{log_{10}N}{log_{10}N+log_{10}(N/(N + 0.4N_\delta ))} \end{aligned}$$

where, N is the length of the sequence and $N_{\delta }$ is the number of sign changes in the sequence.

*Higuchi Fractal Dimension* The Higuchi Fractal Dimension (HFD) is the second implementation of the fractal dimension. HFD is calculated by constructing *k* new small series which are proper subsets of the original series. L is calculated for each of the *k* subsets, and then linear regression is used to find the slope of the graph of L(k) vs ln(1/k), which is the fractal dimension [5, 9, 10].

$$\begin{aligned} L(m,k) = \frac{\sum _{i=2}^{\left\lfloor (N-m)/k \right\rfloor } \left| x_{m+ik}-x_{m+(i-1)k} \right| (N-1))}{\left\lfloor (N-m)/k \right\rfloor k} \end{aligned}$$

#### Hjorth Mobility and Complexity

Derived from the field of signal processing in the time domain, the Hjorth Mobility and Complexity parameters are statistical properties which are normalized slope descriptors [5, 11, 12].

*Hjorth Mobility* Mobility is defined as the square root ratio between the variances of the first derivative of the amplitude. Hjorth proposed this feature as an approximation of the standard deviation of the power spectrum along the frequency axis, or the variation in power in the frequency domain.

*Hjorth Complexity* Likewise, Hjorth also proposed the Complexity parameter as a dimensionless number that is related to the mobility of the first derivative to the mobility of the original EEG signal. The minimum value for the complexity feature can only be derived from a signal which is a perfect sine wave. The complexity measure extracts information on how the EEG signal changes and, more specifically, how unpredictable those changes can be.

#### Entropy

*Spectral Entropy* Spectral entropy is an application of the concept of entropy to the distribution of the Fourier transform and is commonly used in EEG signal processing. It is a method proposed by Rogean Rodrigues Nunes which measures irregularity, complexity or amount of EEG disorders and has been proposed as indicator of anesthetic depth of the signal [5, 8, 10].

$$\begin{aligned} H = -\frac{1}{log(K)}\sum _{i=1}^{K}RIR_i logRIR_i \end{aligned}$$

*SVD Entropy* SVD Entropy is similarly is a measure of the irregularity and complexity of the original signal. The SVD Entropy takes the approach of estimating the number of orthogonal vectors that can define the the dataset within a certain margin. A more complex signal requires more vectors in order to adequately define the signal [5, 8, 10].

#### Fisher information

The Fisher Information metric is another measure of complexity. There are several complexity measures that are computed in different ways because complexity is a subjective measure. Extracting the the most useful information in order to calculate complexity. The periodic and true noise can dominate and obscure any useful information. For this reason, we implement several methods to calculate complexity [5, 10].

$$\begin{aligned} H = \sum _{i=1}^{M-1}\frac{({\bar{\sigma }}_{i+1} - {\bar{\sigma }}_i)^2}{ \bar{\sigma _i}} \end{aligned}$$

#### Detrended Fluctuation Analysis (DFA)

The DFA algorithm quantifies some of the properties of scale-free fluctuations. Scale free in this context is representation of self-similarity where a small section of a larger whole is similar to that whole. A non-stationary stochastic process is said to be self-affine or self-similar in a statistical sense, if a re-scaled version of a small part of its time series has the same statistical distribution as the larger part. For practical purposes, it is sufficient to assess the standard deviation [5, 10].

#### Hurst exponent

The Hurst exponent (H) is also called Rescaled Range statistics (R/S). Similar to the fractal dimension and the Detrended Fluctuation analysis, the Hurst Exponent is also a measure of self similarity and the presence of fractals in the original EEG signal. Again, the EEG signals can be decomposed into smaller components, each one similar to the basic signal. If the Hurst exponent is between 0.5 and 1.0, the signal can be considered to contain self-similar fractals. The Hurst exponent can be closely related to the value of the fractal dimension [5, 10].

$$\begin{aligned} X(t,T) = \sum _{t}^{i=1}(x_i - {\bar{x}}) \end{aligned}$$

where,

$$\begin{aligned} {\bar{x}} = \frac{1}{T}\sum _{i=1}^{T}(x_i), t \epsilon [1..N] \end{aligned}$$

then, the Re-scaled Range Statistics (R/S) is calculated as,

$$\begin{aligned} \frac{R(T)}{S(T)} = \frac{max(X(t,T)) - min(X(t,T))}{\sqrt{(1/T)\sum _{t=1}^{T}[x(t)-{\bar{x}}]^2}} \end{aligned}$$

### Classifier

This section discusses the models used to detect a change in state from PGES to normal activity in EEG signal snippets. This work proposes two classification approaches, one using boosted decision trees and one using a random forest classifier. The training and test set split was performed by randomly choosing 15% of the 134 patients to be in the test set, such that all snippets in the test set are from patients that the model has never seen before to simulate a real-world clinical setting. This train test split was performed 4 times for each trial so as to reduce bias, such that different patients were chosen to be in the test set each time.

Finally, the best models so far were re-trained using a custom coordinate decent algorithm for each respective algorithm in order to tune the associated hyperparameters. Table 2 shows the detailed coordinates used in this analysis.

**Table 2:** XGBOOST hyperparameters used in coordinate decent

Parameter	Values in coordinate
“learning_rate”	[0.05, 0.10, 0.15, 0.20, 0.25, 0.30 ]
“max_depth”	[ 3, 4, 5, 6, 8, 10, 12, 15],
“min_child_weight”	[ 1, 3, 5, 7 ],
“gamma”	[ 0.0, 0.1, 0.2 , 0.3, 0.4 ],
“colsample_bytree”	[ 0.3, 0.4, 0.5 , 0.7, .8, .9, 1, 1.1 ] ,
“eta”	[.3, .2, .1, .05, .01, .005],
“subsample”	[.7, .8, .9, 1, 1.1],
“colsample_bytree”	[.7, .8, .9, 1, 1.1],

*Gradient Boosted Decision Trees* The primary model was chosen to be an implementation of the Gradient Boosted Machine algorithm called XGBOOST. XGBOOST, like all Gradient Boosted Machines, is a weighted sum of many individual decision tree models trained in a gradual, additive and sequential manner. It uses wights to correspond to the importance given to each individual decision tree in the final model. XGBOOST also gives the user the ability to define a custom loss function to relate more appropriately with the real-world application. For the purposes of this project the default loss function is used, but this remains a point of future work, which will be discussed in the discussion section.

*Random Forest Classifier* A second similar model is used in order to analyze the effect of different classifiers on the dataset. The random forest classifier uses the default hyperparameters in Python’s SciKit Learn implementation of the Random Forest classifier.

## Results

The implementation of this machine learning architecture resulted in a max average AUC of 76.02%. In order to vary one variable at a time, the following table is constructed on the default hyperparameters for XGBOOST and Python’s SciKit Learn implementation of Random Forests.

**Table 3:** Classifier AUG results

Classifier	Trial 1 (%)	Trial 2 (%)	Trial 3 (%)	Trial 4 (%)	AVG (%)
*For window size = 4 s*					
XGBOOST	62.77	67.57	75.05	68.90	68.57
Rand Forest	66.09	68.00	71.49	68.90	68.62
*For window size = 7 s*					
XGBOOST	67.82	60.46	63.39	64.29	63.99
Rand Forest	69.17	66.28	71.82	72.18	69.86
*For window size = 10 s*					
XGBOOST	72.21	73.66	70.43	73.66	72.49
Rand Forest	78.58	77.54	70.16	77.80	76.02
*For window size = Random between 1 and 12 s*					
XGBOOST	69.43	66.27	69.43	66.27	67.85
Rand Forest	71.97	71.17	72.58	70.86	71.65
*For window size = Random between 20 and 40 s*					
XGBOOST	66.43	65.72	66.43	65.72	66.08
Rand Forest	65.57	64.62	63.58	64.54	64.58

Table 3 shows the detailed results for each classifier across all trials and the average of all trials. The highest observed AUC score was for the Random Forest Classifier trained on the entire extracted feature space using a EEG snippet length of a constant 10 s. It seems that the window size of 10 s is convenient from a technical point of view in building the model and in a clinical point of view for usefulness.

The feature space that served as the input to the model has a dimension of 180 features and 12.6 million EEG snippets. The feature space was constructed from 18 montages made on 10 raw channels. The breakdown of these features is given by Table 4 and the importance of each feature is tabulated in Table 5.

**Table 4:** Overview of the feature space inputs to XGBOOST

Feature name	Number of columns in feature space
Power Spectral Intensity	10 channels $\times$ 8 scalars
Petrosian Fractal Dimension	10 channels $\times$ 1 scalar
Higuchi Fractal Dimension	10 channels $\times$ 1 scalars
Hjorth Mobility and Complexity	10 channels $\times$ 2 scalar
SVD Entropy	10 channels $\times$ 2 scalar
Spectral Entropy	10 channels $\times$ 1 scalar
Fisher Information	10 channels $\times$ 1 scalar
Detrended Fluctuation Analysis	10 channels $\times$ 1 scalar
Hurst Exponent	10 channels $\times$ 1 scalar
Total	180 features

**Table 5:** Features importance values from XGBOOST

Feature name	Feature importance in resulting model
SVD Entropy	0.090600
Power Spectral Intensity	0.073145
Petrosian Fractal Dimension	0.070548
Hurst Exponent	0.045729
Spectral Entropy	0.031101
Detrended Fluctuation Analysis	0.023219
Higuchi Fractal Dimension	< 0.000001
Hjorth Mobility and Complexity	< 0.000001
Fisher Information	< 0.000001

In order to analyze the feature importance provided by the XGBOOST algorithm, each feature is represented by an average of the measure of importance of all corresponding columns (all its channels). For example the feature Higuchi Fractal Dimension is represented 10 times in the feature space and the resulting importance measure is an average of those 10 columns.

The highest performers were both entropy features, the power spectral intensity, and only the Petrosan Fractal Dimension feature. The lowest performers in the contribution to the model were the Higuchi Fractal Dimension, the Hjorth Mobility and Complexity features and Fisher information.

Finally, details on the hyperparameter sensitivity analysis follows. As discussed previously the hyperparameters were tuned using a coordinate decent algorithm. However, the sensitivity analysis discussed in this section revealed a very low response to changes in the hyperparameters of XGBOOST with frequent local minima, such that for any given starting position in the hyperparameter coordinate space the resulting best algorithm would be very close if not exactly the same as the start position. The greatest change in AUC score from hyperparameter tuning observed was + 1.27%. However the top models did not see an improvement from hyper parameter tuning.

## Discussion

The implementation of a feature space rooted in the fundamental behavior of EEG data as it relates to epilepsy and seizures was successful. The AUC score of 76.02% is satisfactory, considering the possibility of adding more than 10 distinct calculations to the time series data. An interesting point is that similar features calculated in different ways performed very different. For example SVD Entropy was the highest performer while Spectral Entropy was ranked one third the importance. Even more interesting however was the fact that the Petrosan Fractal Dimension was given an importance of 0.0705 while Higuchi Fractal Dimension was given a value of approximately 0.0.

The model’s AUC score was highly dependent on how patients were split into training and test datasets. This shows a potential source of bias in the model implementation that could possibly be addressed with more data from different patients and expanding the feature space to include more common EEG features. The high bias of this method can be addressed also by using a bagging approach to ensemble other automatic methods or classifiers as well as current manual processes in order to create a robust process for detecting the change in state from PGES and normal post seizure activity in patient’s EEG signals.

## Conclusion

Previous work suggests that the duration of PGES is a viable bio marker for predicting a patient’s SUDEP risk. The methods described above are effective at addressing the problem of automatically detecting the end to PGES. A model need not be very complex in order to achieve a high quality of results when special care is given to the inputs to the model. Deploying the solution to a real time system, however, needs to be addressed.

This method can be used in the clinical setting in order to get the duration PGES or validate the duration of PGES that is manually marked by clinicians. This information can then be used in conjunction with other methods to assess the risk a patient has of experiencing SUDEP later in life.

## Data Availability

The data include protected health information, thus are not publicly available.

## Abbreviations

PGES: Post-generalized EEG suppression
SUDEP: Sudden unexpected death during epilepsy
ROC: Receiver operating characteristic curve, a graph showing the performance of a classification model at all classification thresholds
AUC: Area under the ROC Curve
EEG: Electroencephalogram
